# Angioedema of Vermilion Border Lip: A Case Report

**DOI:** 10.7759/cureus.30142

**Published:** 2022-10-10

**Authors:** Karthik Rajaram Mohan, Suresh Chinnakutti, Devaki Murugesan, Sarathchandra Govind Raj, Rajesh Kumar Ganesan

**Affiliations:** 1 Oral Medicine and Radiology, Vinayaka Mission's Sankarachariyar Dental College, Vinayaka Mission's Research Foundation - DU, Salem, IND; 2 Oral and Maxillofacial Surgery, Vinayaka Mission's Sankarachariyar Dental College, Vinayaka Mission's Research Foundation - DU, Salem, IND; 3 Oral Medicine and Radiology, Kanyakumari Government Medical College, Kanyakumari, IND; 4 Prosthodontics, Rajas Dental College, Tirunelveli, IND; 5 Dentistry, Pandian Multispeciality Dental Clinic, Thammampatti, IND

**Keywords:** peanut, angiotensin converting enzyme inhibitors, ige-mediated, c1 esterase inhibitor, angioedema

## Abstract

Angioedema is the diffuse edematous swelling of the soft tissues that most commonly involves the submucosal and subcutaneous connective tissues. It can also affect the connective tissues of the respiratory and gastrointestinal tract. The most common pathogenesis is the degranulation of mast cells, leading to histamine release and increased vascular permeability. The immunoglobulin E (IgE)-mediated hypersensitivity reactions are triggered by foods, preservatives such as sodium benzoate used in peanut butter, dust, drugs like angiotensin-converting enzyme inhibitors (ACE) like captopril, enalapril, lisinopril and pollens, and contact allergies started by prolonged contact with dental rubber dams and cosmetics. Hereditary factors such as quantitative reduction of C1 esterase inhibitor (C1-INH) deficiency and dysfunctional C1 esterase inhibitor (C1-INH) are also postulated in its etiopathogenesis. In addition, lymphoproliferative disorders, bacterial or viral infections, lupus erythematosus, and minor trauma from dental procedures may precipitate an angioedema attack.

## Introduction

Angioedema is described as "circumscribed non-pitting edema affecting the lips, face, neck, and extremities oral cavity, larynx, and stomach" When it affects the larynx, it causes choking due to obstruction of airways, and it can be fatal. In contrast, intestinal angioedema causes stomach pain, interferes with regular bowel movements, and looks like acute appendicitis. Angioedema refers to the swelling of the lower layers of the skin, often around the mouth, or of mucosa or submucosa of the mouth or throat, which can quickly appear in response to an allergen or due to other conditions such as hereditary, C1 esterase inhibitor deficiency or non-functional C1 esterase inhibitor. The allergen can be in the form of seafood, peanut, vaccines, and angiotensin-converting enzyme inhibitor group of drugs used to treat hypertension. Angioedema is marked by a localized increase in blood vessel permeability that causes temporary tissue swelling in deep dermal/subcutaneous tissues, mucosal/submucosal tissues, or both. Bradykinin and mast cell mediators, such as histamine, can cause angioedema. Bradykinin-mediated angioedema can be acquired or hereditary [1-4].

## Case presentation

**Figure 1:**
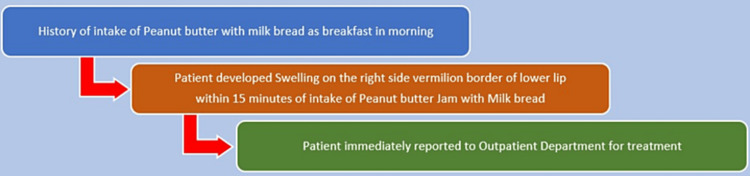
A brief timeline of history

A 24-year-old female reported to the outpatient department with a chief complaint of swelling on her lower lip for the past one day. On eliciting the history of presenting illness, the patient had peanut butter with milk bread as breakfast following the peanut butter consumption; within fifteen minutes, she noted swelling on her right-side vermilion border of the lower lip. Her family history was non-contributory. A general examination revealed no similar swellings on any part of her body. On further clinical examination, there was no cervical lymphadenopathy or fever, and her vitals were stable. She also had no difficulty in breathing, excluding laryngeal involvement. A brief timeline of history is described in Figure 1.

**Figure 2:**
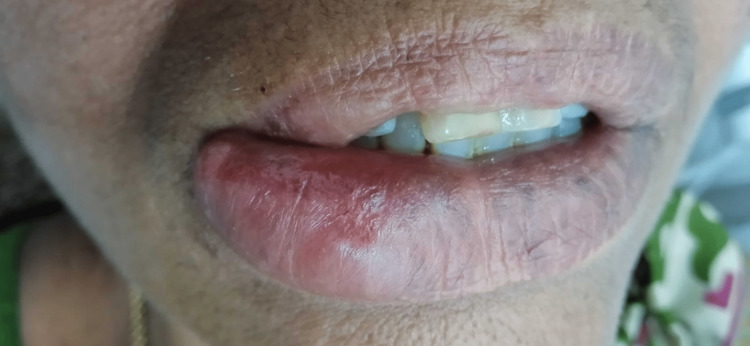
Intraoral examination revealed a diffuse swelling involving the right-side vermilion border of the lip

Extraoral clinical analysis revealed a diffuse swelling in the vermilion border of the lower lip, which was soft, pale, non-tender and measured approximately 3 cm × 2 cm on the right-side vermilion lip border. It was nonpruritic, and no evidence of erosion or ulceration was noted (Figure 2).

**Figure 3:**
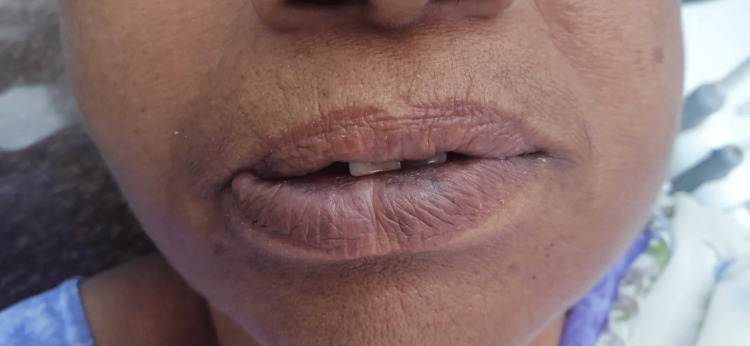
Follow-up post-treatment photograph after one week showed complete resolution of the swelling on vermilion border of the lower lip

On palpation, the swelling was soft in consistency, non-pitting and non-tender. Correlating the history of a diffuse swelling on the vermilion border of the lip with a history of sudden onset in an hour, and no record of any trauma or fever, a provisional diagnosis of allergic cheilitis was made. The following medications were prescribed for the swelling oral tablet Levocetirizine 5 mg twice daily. In addition, tablet Fexofenadine 60 mg twice daily for seven days. Finally, the Hydroxyzine pill was prescribed at 25 mg only at night for one week. The patient was recalled for follow-up after seven days. The diffuse swelling on the vermilion border of the lip subsided after she took the prescribed medication. The patient was placed for follow-up clinical examination after a week of prescribed medications, which resulted in complete resolution of the lesion on the vermilion border of the right-side lower lip (Figure 3).

Differential diagnosis

The differential diagnosis of swelling involving lips includes cheilitis glandularis, actinic cheilitis, post-traumatic swelling and angioedema. Cheilitis glandularis involves entire and diffuse involvement of the upper or lower lip due to inflammation of minor salivary glands in the vermilion border of the lip, which was not noted in our case, as in contrast in our case, only the right half of the vermilion border of the lower lip is involved. Actinic cheilitis involves inflammation of the lip following harmful Ultraviolet (UV) exposure to sun rays. No history of such disclosure to sun rays exists, as she works as a housemaid indoors. Post-traumatic swelling consists of the swelling of the lip followed by trauma. In our case, no history of trauma to the lip exists.

Investigations

**Figure 4:**
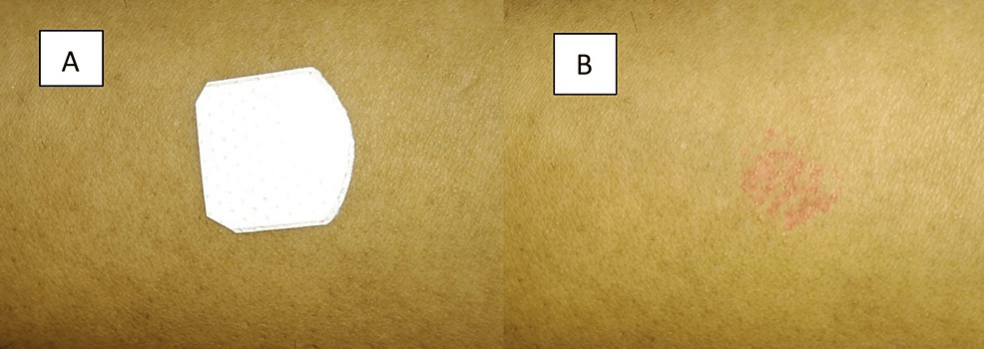
(A, B) Patch test revealed skin reactions of erythema after 48 hours on the site of the applied patch on the flexor aspect of patient's left forearm

A Food diary was prepared in which a chronological listing of all her food intakes is listed, which revealed that whenever the patient intakes peanut butter, she develops a sudden transient swelling of her lips. Sodium benzoate, the most commonly used preservative in peanut butter to retain its freshness, has caused angioedema in our case, which was confirmed by a patch test (Figures 4A, 4B). Hence, a final diagnosis of peanut butter-induced angioedema of the lip was made.

## Discussion

Definition

Angioedema is a circumscribed non-pitting edema of submucosal or subcutaneous tissues affecting the lips, face, eye, oral cavity, larynx, and gut. Angioedema affects the lip or face and is most frequently disfiguring. The face, lips, tongue, throat, supraglottic, and subglottic regions are the most often affected body parts. Along with the mucous membranes of the gastrointestinal tract and the genitalia, angioedema can also affect the hands and feet [1].

History

In 1876, J. L. Milton was the first to describe HAE, and in 1882, Quincke referred to the condition as “angioneurotic edema.” The adjective “neurotic” was added to the disease's name since it was found that mental stress could cause exacerbations of the condition. The first person to describe HAE in full across five generations was Sir William Osler in 1888, who also noted the illness' hereditary nature. A few decades later, Donaldson and Evans published their research on the biochemical cause of HAE - the lack of C1-INH [1].

Classification of angioedema

Angioedema is classified based on its diversified etiologies as hereditary, acquired, drug-induced, or unknown cause (idiopathic) (Table 1).

**Table 1:** Types of angioedema based on etiologies

Types of Angioedema	Etiology
Allergic Angioneurotic edema (histaminergic acquired angioedema)	Occurs in association with anaphylaxis
Non-allergic Angioneurotic edema (non-histaminergic angioedema)	Occurs isolated or in combination with urticaria
Hereditary angioneurotic edema	Type 1 angioneurotic edema (lack of C1 inhibitor molecule)
	Type 2 angioneurotic edema (dysfunctional C1 inhibitor molecule)
Acquired angioneurotic edema	Exposure to heat/cold, Bacterial or virus infections, vaccines, lymphoproliferative disorders, autoimmune disorders, neoplasia, trauma, e.g., dental work, surgery, vaccines.
Drug-induced angioneurotic edema	Angiotensin Converting enzyme inhibitors, angiotensin-2 antagonists, oral contraceptive pills, non-steroidal anti-inflammatory drugs, Proton pump inhibitors, selective serotonin reuptake inhibitors
Idiopathic angioneurotic edema	Unknown etiology

Epidemiology 

Angioedema is transmitted as a rare autosomal dominant condition affecting one in 10,000 to 1:150,000 individuals. Females are affected more commonly than males [3].

Etiopathogenesis

Genetics

Three-quarters of HAE patients have mutations in the C1-INH gene on chromosome 11 that is inherited in an autosomal dominant form; in the other one-fourth of HAE patients, the mutation arises spontaneously. Therefore, the diagnosis of HAE should not be disregarded even without a family history. The initial complement system component, C1-INH, is inhibited by this mutation, which also results in the inactivation of the coagulation factors XII, XIIa, and XIa, as well as the direct suppression of activated kallikrein. Since HAE type 3 has been linked to an autosomal dominantly inherited gain-of-function mutation in the coagulation factor XII, it is no longer thought to be an X-linked disease. There may also be undiscovered mutations that impact the regulation of the kinin-kallikrein system. Additionally, estrogen-containing oral contraceptives or hormone replacement treatment, which may affect the kinin pathway, have also been linked to this particular kind of HAE [4].

The biological function of complement inhibitor C1-INH in the complement system is to inhibit C1 autoactivation by dissociating the C1q subunit and binding to C1r and C1s. This interaction keeps the Classical route inert because it creates an inactive C1r2-Cs2-(C1-INH)2 complex that cannot cleave and activate complement components C2 and C4. As a result, in HAE with low or absent C1-INH, the early complement cascade (C1, C2, and C4) is unchecked and activated even before other inhibitors (C4-binding protein and factor I) can stop the pathway, which results in the consumption of the complement factors (C4) and increased production of anaphylatoxins (C3a, C5a), chemotaxins (C3b), and localized edema of the skin. Coagulation factors, XIIa and its metabolite XII f, and the direct suppression of active kallikrein, are crucially inactivated by the C1-INH. Therefore, factors XIIa and XIIf can produce much higher amounts when reduced CI-INH levels and activity. Factor XII is activated by factor XIIa, and factor XII, in turn, activates more molecules of factor XIIa. The significant rise in factor XIIa levels results from the positive feedback loop's unopposed strengthening. Additionally, factor XIIa converts prekallikrein to the active enzyme kallikrein, and kallikrein then breaks down high-molecular-weight plasma kininogens, causing an excessive amount of bradykinin to be released [3].

A consequence of diminished C1-INH activity is the loss of its direct inhibitory influence on kallikrein activity. Kallikrein cleaves high-molecular-weight plasma kininogens to release bradykinin, further boosting bradykinin synthesis. Bradykinin is a contact system mediator that binds to Bradykinin type 2 receptors on endothelial cells, increasing vascular permeability (resulting in edema, swelling, and ascites), vasodilation (causing congestion, erythema, and hypotension), and contraction of nonvascular smooth muscle. As a result, in the absence of CI-INH production in HAE, bradykinin is generated unchecked (cramps, spasms, and pain) [3].

A serine protease inhibitor is a C1 inhibitor (SERPIN). SERPING 1 mutation or mutant genes that encode for bradykinin-metabolizing and active enzymes cause C1 inhibitor deficiency. Plasma levels of HAE type 1 are affected by an abnormal build up of C1 inhibitors in dominant dangerous diseases. Secondary causes of HAE include mutations in the F12 gene, angiopoietin-1, plasminogen, or unidentified genes [4].

Histamine from mast cells, bradykinin, C1 inhibitor deficiency

The most frequent type of angioedema, mediated by histamine, results from basophil and mast cell activation. Angioedema is mediated by bradykinin (HAE, acquired C1-inhibitor deficiency, and ACE inhibitor-associated angioedema). Hives and allergic responses do not cause this illness. The C1-inhibitor regulates complement and the contact system; if it is lacking or dysfunctional, it activates the contact system, resulting in runaway production of kallikrein, which causes the proteolysis of high-molecular-weight kininogen and bradykinin, which causes swelling by increasing vascular permeability. Angioedema is mediated by bradykinin, produced excessively when there is a lack of a C1 inhibitor-angioedema related to ACE inhibitor: decrease in bradykinin degradation. Bradykinin and histamine promote regional microvascular permeability. Cyclooxygenase 1 inhibitor influences the metabolism of arachnoid acids, leukotriene/prostaglandin binding to the receptor, or Immunoglobulin E (IgE)-mediated when a non-steroidal Anti-inflammatory Drug (NSAID) is taken [4].

Clinical features

Although there can be a significant amount of inter- and intra-individual symptom variability among HAE patients, episodes of marked, diffuse, and recurrent edema are the hallmarks of the condition. These episodes typically follow a typical pattern of gradual progressive swelling over the first 24 hours, followed by slow symptom resolution over the following 48-72 hours. All skin layers, as well as layers of the walls of solid organs and hollow visceral organs, are affected by the swelling. Angioedema of the lip can be lateralized to one-half in rare instances. Cutaneous edema most frequently affects the face, extremities, and genitalia and is non-pitting, non-urticarial, and has ill-defined boundaries. Approximately 80% of patients experience facial edema, which includes the lips, mouth, oropharynx, and periorbital tissues. Extremity swelling is also highly prevalent and can affect considerable sections of the arms or legs. It can start asymmetrically and progress over a few hours or days. Patients may endure extreme discomfort if the swelling is moderate to severe in delicate locations like the face or urogenital regions [4].

The digestive system is frequently affected by HAE, leading to bowel angioedema, which can be exceedingly severe and sometimes mistaken for an acute abdomen due to its lack of cutaneous signs. Typical symptoms include nausea, vomiting, and, less frequently, diarrhea. This wide range of acute abdominal symptoms may prompt many emergency room trips and necessitate exploratory abdominal surgery, cholecystectomies, and appendectomies [4].

Despite being much less frequent than cutaneous or abdominal involvement, laryngeal edema can cause death in HAE due to the risk of asphyxiation from upper-airway constriction. Mortality rates in undetected instances might reach 30% to 40%. Early symptoms may include a lump or feeling of tightness in the throat, minor voice changes, or dysphagia, which may progress to dyspnea due to airway obstruction. Laryngeal edema episodes linked with HAE typically advance gradually over several hours. However, a quicker transition from the onset to airway blockage cannot be ruled out [4].

HAE attacks are typically spontaneous and unprovoked. However, they can be brought on by local tissue trauma (such as from dentistry and medical operations), mental stress, menstruation, oral contraceptives, infections, or drugs like ACE inhibitors like Enalapril and Perindopril. Furthermore, HAE attacks are extraordinarily unpredictable and varied, making patients and caregivers anxious and concerned [4].

HAE manifests as recurring episodes of swelling or stomach pain and starts in childhood or young adulthood before worsening throughout puberty. Prominent prodromal signs like erythema marginatum can appear in patients (erythematous, serpentine, non-pruritic rash). An acute attack lasts two to three days to end after peaking on the first day. Comparable to HAE, acquired C1 inhibitor deficiency exhibits similar symptoms. However, the low complement C1 inhibitor is frequently caused by an underlying lymphoproliferative disease that raises protein consumption and an antibody against C1-INH that causes an excess of bradykinin. Bradykinin-associated angioedema has the following characteristics: longer duration, increased severity of clinical symptoms and is not associated with urticaria. Therefore, a thorough clinical examination of the head, neck, and abdomen and monitoring of vital signs is essential [4].

Diagnostic aids for angioedema in other regions

Zvidi et al. stated the use of capsule endoscopy in the diagnosis of Tacrolimus-induced intestinal angioedema [5]. Vallabh et al. stated the use of computed tomography in the diagnosis of small bowel obstruction caused by angioedema induced by ACE [6].

Laboratory tests for angioedema

The various laboratory tests for angioedema are described in Table 2.

**Table 2:** Laboratory tests and their indications for angioedema

Laboratory tests	Indication
In vivo allergic skin testing-Patch test	suspected allergens in cosmetics
Invitro RAST ( Radioallergosorbent test )	allergen-specific foods,hymenoptera (wasp,bee, fire ant) venom
C1s -binding enzyme-linked immunosorbent assay (ELISA)	estimation of C1 esterase inhibitor levels
Provocation tests	Bronchoprovocation tests – serial determination of peak expiratory flow rate using portable peak flowmeter during exposure to suspected airborne allergen. Oral provocation tests
Elevated Serum Tryptase / Baseline Serum Tryptase (BST) levels	Tryptase is a mast cell -derived neutral protease Has more half-life of 60-90 minutes than histamine which has a very short half life of 1 minute 4 seconds (102 seconds)
Serum C4 Levels	For hereditary angioedema
C1 esterase inhibitor deficiency	For hereditary angioedema
Potential Biomarkers for angioedema	C1, C1N -INH complex,antithrombin (AT) complex, Ficolin 3, Bradykinin, Plasma amidase activity, Aminopeptidase (APP), Carboxypeptidase N (CPN), Dipeptidyl peptidase IV (DPPIV), activated partial thromboplastin time (aPTT), D-dimer, Von-willebrand factor, Adrenomedullin, Atrial natriuretic peptide, Vascular endothelial Growth Factor (VEGF), Angiopoietin 1 and 2, C-Reactive protein (CRP), soluble E-selectin, Erythrocyte sedimentation rate (ESR), Neutrophil, Tumour necrosis factor-alpha (TNF-α), Progesterone, Sex Hormone binding Globulin (SHBG), Interleukin 8,17, Granulocyte colony stimulating factor ( G-CSF), Granulocyte macrophage colony stimulating factor (GMCSF), Myeloperoxidase, Neutrophil elastase. Endocan – a novel potent endothelin marker of endothelial dysfunction in C1- inhibitor deficiency hereditary angioedema

Therapeutic modalities for angioedema

Treatment modalities for angioedema are described in Table 3 [3,5-15].

**Table 3:** Therapeutic modalities for angioedema

H 1 antihistamines	Hydroxyzine 10 mg twice daily to 25 mg thrice daily or 50-75mg at night
	Cyproheptadine 4 mg four times daily
	Fexofenadine – 60 mg twice daily Loratidine – 10 mg per day Cetirizine – 10 mg per day
	Diphenhydramine 25 – 50 mg orally, IM or IV every 4-6 hours
Tricyclic antidepressants	Doxepin – 25 -75 mg at night
Systemic Corticosteroids	Prednisolone 40 mg per day
	Cyclosporine 3-5 mg per kg /day
	Epinephrine 1 : 1000 0.2-0.5 ml injected intramuscularly in the anterolateral thigh
Bronchodilator	Aminophylline 0.5 mg /kg/h IV with loading dose of 6mg/kg over 30 minutes
Hypotension	Clonidine 0.5 -1 mg at bed time, Phenylephrine 0.1-0.5 mg IV every 10-15 minutes. Maximum dose : 0.5 mg IV, 2-5 mg SC /IM every 2 hours followed by 1-10 mg maintenance dose. Severe Hypotension : 100-150 mcg IV bolus every 5-10 minutes, Maximum dose 500 mcg or 100-180 mcg/ min or 0.5 mcg/kg/min (IV infusion ) Digoxin : IV: 8-12 mcg/kg (0.008-0.012 mg/kg) total loading dose; administer 50% initially; then may cautiously give 1/4 the loading dose q6-8hr twice; perform careful assessment of clinical response and toxicity before each dose PO: 10-15 mcg/kg total loading dose; administer 50% initially; then may cautiously give 1/4 the loading dose q6-8hr twice; perform careful assessment of clinical response and toxicity before each dose Maintenance PO: 3.4-5.1 mcg/kg/day or 0.125-0.5 mg/day PO; may increase dose every 2 weeks based on clinical response, serum drug levels, and toxicity IV/IM: 0.1-0.4 mg qDay; IM route not preferred due to severe injection site reaction
Icatibant (Firazyr@)	Synthetic decapeptide used in treatment of acute angiotensin-converting enzyme inhibitor angioedema Bradykinin B2 receptor antagonist 30 -45 mg three times a day for every 6 hours
Ecallantide (Kalbitor@)	30 mg subcutaneous
Lanadelumab (TAKHZYRO)	300 mg / 2ml injection Subcutaneous for every 2 weeks or 1000 Units IV every 3-4 days
Mephenamic acid, Tranexamic acid	250 mg, 500 mg Tranexamic acid
Danazol	200 mg twice or thrice daily
Stanazol	2 mg daily
Plasma derived nanofiltered C1 INH (Berinert,CSL Behring)	20 U /Kg IV
Recombinant Human C1 INH ( Ruconest, Pharming)	50 U/ Kg .Upto 4200 U IV
Gene therapy	Adenoassociated (AAO) virus
Venom immunotherapy	(Hymenoptera and vespid venom )Mixed venom dose (300 mcg) – more protection than Hymenoptera Single venom dose (50 mcg). Maintenance dose every 4 weeks or atleast once a year

Special considerations for pregnant and pediatric patients with angioedema

The suggested treatment during pregnancy is a plasma-derived nano-filtered complement C1 inhibitor; however, in acute episodes, bradykinin receptor antagonist Icatibant may be used because it is risk-free and has no adverse effects on the mother or the fetus. Pediatric patients are given doses of 500 units (10-25 kg), 1,000 units, and 1,500 units when they weigh more than 25 kg. Pediatric patients younger than 12 can safely and effectively use plasma-derived complement C1 esterase inhibitors. Icatibant, a well-tolerated drug for children, may be used to treat angioedema brought on by angiotensin II receptor blockers. A three-day sustained response is shown when treating acute HAE with recombinant human C1 esterase inhibitor. The immediate treatment of pancreatitis caused by HAE is managed with a complement C1 inhibitor. Patients with life-threatening orolingual angioedema respond quickly to Icatibant therapy after receiving recombinant tissue plasminogen activator infusion. Angiotensin-converting enzyme inhibitors and dipeptidyl peptidase-4 inhibitors should not be used concurrently because dipeptidyl peptidase-4, like an angiotensin-converting enzyme, is a critical enzyme in the breakdown of bradykinin-utilization of liquid steroids in individuals with severe angioedema and urticaria combined with severe dysphagia due to anaphylaxis. Use of the rare condition idiopathic non-histaminergic acquired angioedema, which is resistant to antihistamines and omalizumab (anti-immunoglobulin-E antibody), is given subcutaneously in dosages of 300 mg for a period of 12 to 24 weeks [16].

Novelty

The novelty of this case report is that the swelling occurred lateralized only on the right side of the vermilion border of the lip and was diagnosed by careful history taking and supportive investigations like food charting and patch test. Early intervention of angioedema was done, thereby dreadful life-threatening complication like difficulty in breathing that required tracheostomy procedure was prevented in this case.

Limitations

The laboratory parameters and specific novel biomarkers like endocan were not assayed in this case to rule out the hereditary type of angioedema.

## Conclusions

Angioedema is a rare disease that results in intense, transient swelling causing disfigurement of a localized body area involving the oral mucosa, skin, and subcutaneous tissues involving the face, lips, pharynx, tongue, supraglottic areas, hands, feet, and genitalia. The sudden onset of swelling on the vermilion border of the lip must not be overlooked by the dentist and leads to misdiagnosis and delay in diagnosis, can result in death and must be attended promptly on time under emergency in the Intensive care unit, as it causes complications like obstruction of airways resulting in choking due to swelling of tongue, pharynx, larynx, lips. It is, therefore, essential to monitor and access vital signs during an attack of angioedema. It is also essential to follow up and evaluate such patients closely to avoid life-threatening complications in the future.
